# Association between *Toxoplasma gondii* infection and psychiatric disorders: a cross-sectional study in China

**DOI:** 10.1038/s41598-022-16420-y

**Published:** 2022-09-05

**Authors:** Taixiu Liu, Peng Gao, Deyun Bu, Dong Liu

**Affiliations:** 1 Department of Clinical Laboratory, Shandong Daizhuang Hospital, Jining, 272051 Shandong People’s Republic of China; 2 Department of Clinical Laboratory, Qingdao Sanatorium of Shandong Province, Qingdao, 266071 Shandong People’s Republic of China; 3 Department of Clinical Laboratory, Affiliated Hospital of Jining Medical University, Jining, 272029 Shandong People’s Republic of China

**Keywords:** Biomarkers, Risk factors

## Abstract

Psychiatric patients have become the focus of public attention, and current research suggests a possible link between *Toxoplasma gondii (T. gondii)* infection and mental illness. To understand the current situation of *T. gondii* infection in psychiatric patients in the study area, the relationship between *T. gondii* infection and mental diseases, and the influence of *T. gondii* infection on psychiatric patients, this study examined 3101 psychiatric inpatients from 2015 to 2020. All people included in the study were tested for anti-*Toxoplasma* IgM antibody and anti-*Toxoplasma* IgG antibody. Additionally, 4040 individuals from the general population were included as controls. The chi-square test and logistic regression analysis were carried out to determine the association between psychiatric disorders and *T. gondii* infection. The seroprevalence of anti-*Toxoplasma* IgM antibody was 0.23% (7/3101) in psychiatric inpatients and 0.11% (2/1846) in the general population, and there was no significant difference (*p* > 0.05). The seroprevalence rate of anti-*Toxoplasma* IgG antibodies was 3.03% (94/3101) in psychiatric inpatients and 1.05% (23/2194) in the general population, and there was a significant difference (*p* < 0.01). The seroprevalence of anti-*Toxoplasma* IgG antibody in psychiatric inpatients was significantly different between different age groups (*p* < 0.01). The positivity rate of anti-*Toxoplasma* IgG antibodies was 5.17% (3/58) in patients with mania, 3.24% (8/247) in patients with recurrent depressive disorder, 3.54% (13/367) in patients with depression, 3.22% (39/1213) in patients with schizophrenia, 2.41% (18/748) in patients with bipolar disorder and 2.25% (2/89) in patients with dissociative disorder. Compared to the general population, patients with mania (OR = 5.149 95% CI 1.501–17.659 *p* = 0.009), schizophrenia (OR = 3.136 95% CI 1.864–5.275 *p* = 0.000), depression (OR = 3.466 95% CI 1.740–6.906 *p* = 0.000), recurrent depressive disorder (OR = 3.160 95% CI 1.398–7.142 *p* = 0.006) and bipolar disorder (OR = 2.327 95% CI 1.249–4.337 *p* = 0.008) were found to be significantly associated with the seroprevalence of anti-*Toxoplasma* IgG antibody. This study suggests that the seroprevalence of *T. gondii* infection in psychiatric patients was higher and that age was an influencing factor of *T. gondii* infection in psychiatric patients. *T. gondii* infection was associated with mania, schizophrenia, depression, recurrent depressive disorder and bipolar disorder.

## Introduction

*T. gondii* is an obligate intracellular parasite with a worldwide distribution, and it is widely parasitic on the nucleated cells of humans and animals. *T. gondii* causes toxoplasmosis and infects about one third of the world's population^1^. This parasite is particularly common in developing countries, but it is also present in developed countries. Cats are the definitive hosts of *T. gondii*, while human, birds and reptiles are the intermediate hosts. *T. gondii* is mainly parasitic in the host's brain and muscle tissues, and *T. gondii* infection in humans is mainly caused by ingesting water and food contaminated by *T. gondii* or through vertical transmission from mother to child. When *T. gondii* infects people, it forms tissue cysts in some parts of the body, and these cysts can be reactivated when the immune function is weak^2^. Numerous studies suggest that *T. gondii* can alter human behaviour and increase its impact on public health^3^. Many studies have found a higher serological prevalence of *T. gondii* in psychiatric patients, which suggests that there may be a correlation between *T. gondii* infection and psychiatric disorders^4^. Current studies have focused on possible associations with *T. gondii* infection in schizophrenia, bipolar disorder and depression, but there are wide variations^5^. For example, studies have reported the seroprevalence of *T. gondii* among patients with schizophrenia (50.9%) and patients with bipolar disorders (52.6%)^6^. Other studies have reported that the anti-*T. gondii* IgG seropositivity rate was 18.8% among patients with bipolar disorders^7^.

The present study examined antibodies against *T. gondii* in psychiatric patients to determine the prevalence of *T. gondii* infection in psychiatric patients in the study area, identify the association between *T. gondii* infection and psychiatric disorders, and provide a theoretical basis and data-based support for the health administrative department in the region to formulate relevant prevention and control strategies for *T. gondii* infection among psychiatric patients.

## Method

This was a cross-sectional study designed to assess the prevalence of *T. gondii* infection in psychiatric patients in the study area. Serum samples were collected from newly admitted psychiatric patients from 2015 to 2020 in Shandong Daizhuang Hospital, and the corresponding medical records were collected at the same time. This study obtained the informed consent of all participants and/or their legal guardians, as reflected in the medical records and medical orders. The inclusion criteria for psychiatric patients were as follows: (1) newly admitted inpatients, (2) diagnosed by a psychiatrist as mentally ill according to the *International Classification of Diseases, 10th Revision*, o*r The Diagnostic and Statistical Manual of Mental Disorders (DSM–5)*, and (3) available medical records. The exclusion criteria were as follows: patients with mental disorders due to alcohol/drug intoxication or abstinence, neurodevelopmental disorders, or traumatic and stress-related disorders. The doctor informed the patient according to the diagnosis and treatment process and issued a test application for anti-*Toxoplasma* antibodies. The patients were sent to the medical laboratory to provide blood samples. A total of 3101 psychiatric patients were included.

Additionally, a total of 4040 serum anti-T*oxoplasma* antibody test results were collected from individuals without psychiatric disorders at the Genetic Counselling Clinics and Physical Examination Center of the Affiliated Hospital of Jining Medical University or other companies. This included 1846 tests for anti-*Toxoplasma* IgM antibody and 2194 tests for anti-*Toxoplasma* IgG antibody. For privacy and other reasons in the general population, we only collected information about their age, except for *Toxoplasma* antibody test results. Participants in the study ranged in age from 9 to 77, the average age of psychiatric patients included in the study was 32.85 ± 10.76 years old, the average age of the general population tested for anti-*Toxoplasma* IgM antibodies was 32.33 ± 5.68 years old, and the average age of general people tested for anti-*Toxoplasma* IgG antibodies was 32.3 ± 5.77 years old.

This study collected medical records of inpatients with mental illness for further research. The positivity rate of anti-*Toxoplasma* IgM antibody was too low to perform an accurate statistical analysis; therefore, we analysed only the epidemiological characteristics of anti-*Toxoplasma* IgG antibody. Based on the collected medical records, the psychiatric inpatients were divided into a female group and a male group, with males accounting for the majority. The inpatients were divided into 3 groups based on age: 9–20 years old, 21–40 years old, and 41–77 years old. Han was the most common ethnicity, and individuals of other ethnicities accounted for approximately 1% of the sample. Marital status was categorized as single, married or divorced. According to their residence and surroundings, the inpatients were divided into rural and urban groups. Patients were also divided into six groups based on their occupation: jobless, farmer, worker, student, cadre and other groups. The population included in this study mainly came from Jining, Heze, Zaozhuang, Taian, Linyi and other regions, covering an area of approximately 53,000 square kilometres with a population of approximately 37.5 million. The terrain of the region includes plains, hills and mountains. According to the severity of mental illness, the inpatients were divided into a general group and a severe group. The severe group included patients with schizophrenia, schizoaffective disorder, bipolar disorder or other psychiatric disorders (excluding any substance-induced psychosis and unspecified psychosis) according to the *International Classification of Diseases, 10th Revision* or *The Diagnostic and Statistical Manual of Mental Disorders (DSM–5)*. Based on existing research results classification and the type of mental illness diagnosed, people with mental illness are divided into seven groups: schizophrenia, bipolar disorder, depression, recurrent depressive disorder, dissociative disorder, mania and others (Table 1).

**Table 1:** Demographic characteristics of the psychiatric patients ( N= 3101).

Variables		Number	Percentage (%)
Sex	Male	2535	81.75
	Female	566	18.25
Age (years)	9–20	427	13.77
	21–40	1942	62.62
	41–77	732	23.61
Ethnicity	Han	3072	99.06
	Others	29	0.94
Marital status	Single	1252	40.41
	Married	1701	54.91
	Divorced	145	4.68
Occupation	Jobless	1288	42.09
	Farmer	908	29.67
	Worker	223	7.29
	Student	329	10.75
	Cadre	119	3.89
	Other	193	6.31
Residence	Rural	2262	73.20
	Urban	828	26.80
Region	Jining	2095	67.60
	Heze	430	13.88
	Zaozhuang	168	5.42
	Taian	155	5.00
	Linyi	87	2.81
	Other parts of Shandong	59	1.90
	Other provinces	105	3.39
Severity	General	1093	35.25
	Severe	2008	64.75
Psychosis type	Bipolar disorder	748	24.12
	Schizophrenia	1213	39.12
	Depression	367	11.83
	Mania	58	1.87
	Recurrent depressive disorder	247	7.97
	Dissociative disorder	89	2.87
	Others	379	12.22

When patients arrived at the medical laboratory, they were informed of the purpose of drawing blood. Then, 5 ml of fasting blood was drawn from the subjects. The sample was centrifuged at 4000 r/min for 10 min, and then, the serum was separated for testing. Serological marker tests of *T. gondii* infection include anti-*Toxoplasma* IgM and IgG antibody measures using enzyme-linked immunosorbent assay (ELISA) (Auto Bio, China).

Statistical analysis was performed with SPSS v22 software (IBM Inc., Chicago, USA). Demographic characteristics were assessed with descriptive analysis. Pearson’s chi-square test, continuity correction, or Fisher's exact tests were performed for the analysis of categorical data. The putative risk factors were indicated by odds ratios (ORs) with 95% confidence intervals (95% CIs) using logistic regression, and a *p value* of < 0.05 was considered statistically significant.

### Ethics approval and consent to participate

The research protocol was approved by the ethics committee of Shandong Daizhuang Hospital, and all experiments were performed in accordance with the Declaration of Helsinki and relevant guidelines and regulations.

## Results

As serological markers of *T. gondii* infection, anti-*Toxoplasma* IgG antibody presents latent exposure, and anti-*Toxoplasma* IgM antibody presents acute/recent exposure. The positivity rate of anti-*Toxoplasma* IgM antibody in psychiatric patients was 0.23% (7/3101), while in the general population, it was 0.11% (2/1846), with no statistically significant difference between the two groups (*p* = 0.359). The positivity rate of anti-*Toxoplasma* IgG antibody in psychiatric patients was 3.03% (94/3101), while in the general population, it was 1.05% (23/2194). There were statistically significant differences between the two groups, and psychiatric patients had a higher positivity rate than the general population (OR = 2.951, 95% CI 1.864–4.671, *p* = 0.000) (Table 2).

**Table 2:** Positive rate of anti-*Toxoplasma* IgG antibodies and anti-*Toxoplasma* IgM antibodies.

Antibodies	Number	Positive number (Percentage %)	*χ*2 -value	OR	95% CI	*p value*^c^
**Anti-IgG**						
General population	2194	23 (1.05)	23.382^a^	1		
Psychiatric patients	3101	94 (3.03)		2.951	1.864–4.671	0.000
**Anti-IgM**						
General population	1846	2(0.11)	0.351^b^	1		
Psychiatric patients	3101	7 (0.23)		2.086	0.433–10.052	0.359

^a^Pearson’s chi-square test, ^b^Continuous calibration chi-square test, ^c^Binary logistic regression analysis.

Through further analysis of anti-*Toxoplasma* IgG antibody in psychiatric patients, the results showed that there were significant differences in the positivity rates of anti-*Toxoplasma* IgG antibody among different age groups (*χ*2 = 12.234, *p* = 0.002). Compared with the 41–67 and 9–20 age groups, the 21–40 age group had the lowest positivity rate of anti-*Toxoplasma* IgG antibody, indicating that this age range was a potential protective factor (OR = 0.437, 95% CI 0.266–0.720, *p* = 0.001). There was no statistically significant difference in the positivity rates of anti-*Toxoplasma* IgG antibody in patients with various types of mental illness. There was no significant difference in the positivity rate of anti-*Toxoplasma* IgG antibody in the sex, ethnicity, marital status, residence, and severity groups (Table 3).

**Table 3:** Analysis of the seroprevalence of anti-*Toxoplasma* IgG antibody in psychiatric patients (*N* = 3101).

Variables		*N*	Positive (*n*)	Positivity rate(%)	*χ*2 value	OR	95% CI	*p value*^c^
Sex	Female	566	23	4.06	2.510^a^	1		
	Male	2535	71	2.80		0.647	0.378–1.109	0.113
Age(years)	9–20	427	13	3.04	12.234^a^**	0.679	0.256–1.798	0.436
	21–40	1942	45	2.32		0.437	0.266–0.720	**0.001**
	41–77	732	36	4.92		1		**0.004**
Ethnicity	Han	3072	93	3.03	0.000^b^	1		
	Others	29	1	3.45		1.392	0.180–10.759	0.751
Marital status	Single	1252	33	2.64	2.375^a^	1		0.407
	Married	1701	54	3.17		0.981	0.540–1.781	0.950
	Divorced	145	7	4.83		1.715	0.689–4.266	0.246
Occupation	Jobless	1288	46	3.57	3.308^a^	1.00		0.509
	Cadre	119	3	2.52		0.586	0.165–2.085	**0.409**
	Farmer	908	27	2.97		0.830	0.508–1.356	0.456
	Worker	233	6	2.69		0.660	0.254–1.714	0.393
	Student	329	8	2.43		0.598	0.221–0.619	0.312
	Others	193	3	1.55		0.339	0.099–1.164	0.086
Residence	Urban	828	23	2.78	0.268^a^	0.984	0.550–1.762	0.957
	Rural	2262	71	3.14		1		
Region	Jining	2095	61	2.91		1		0.697
	Heze	430	14	3.26		1.096	0.600–2.000	0.766
	Zaozhuang	168	7	4.17	6.392^a^	1.547	0.687–3.485	0.292
	Taian	155	5	3.23		1.188	0.464–3.042	0.720
	Linyi	87	1	1.15		0.414	0.056–3.048	0.386
	Other parts of Shandong	59	0	0.00		0.000		0.997
	Other provinces	105	6	5.71		1.999	0.773–5.175	**0.153**
Severity	Severe	2008	58	2.89	0.395^a^	0.837	0.101–6.901	0.869
	General	1093	36	3.29		1		
Psychosis type	Bipolar disorder	748	18	2.41	2.608^a^	1		0.798
	Schizophrenia	1213	39	3.22		1.325	0.744–2.360	0.339
	Dissociative disorder	89	2	2.25		0.570	0.043–7.649	0.671
	Depression	367	13	3.54		1.085	0.116–10.160	0.943
	Recurrent depressive disorder	247	8	3.24		0.913	0.094–8.903	0.937
	Mania	**58**	**3**	**5.17**		**1.901**	0.162–22.311	0.609
	Others	379	11	2.90		0.865	0.111–6.741	0.890

^a^Pearson chi-square test. ^b^Fisher's exact probability method. ^c^Binary logistic regression analysis. **p < 0.01.

**Figure 1:**
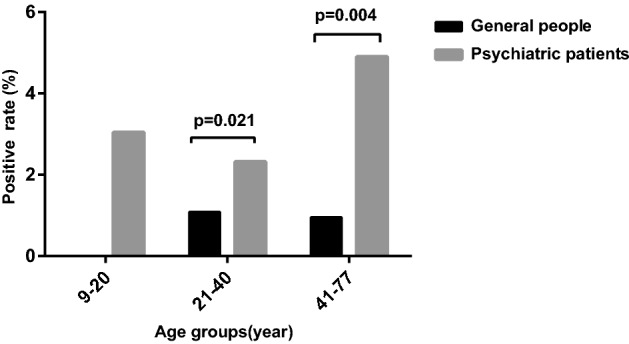
Comparison of anti-*Toxoplasma* antibody-positivity rates between psychiatric patients and the general population in different age groups. Compared with the general population, psychiatric patients in the 21–40 years old age group (OR = 2.174 95% CI 1.290–3.664, *p* = 0.004) and 41–77 years old age group (OR = 5.405 95% CI 1.291–22.637 *p* = 0.021) had a higher positivity rate of anti-*Toxoplasma* IgG antibody.

The positivity rate of anti-*Toxoplasma* IgG antibody in the general population was 0.00% (0/37) in the 9–20 years old age group, 1.08% (21/1946) in the 21–40 years old group and 0.95% (2/211) in the 41–77 years old group. The positivity rate of anti-*Toxoplasma* IgG antibody in the psychiatric patients was 3.04% (13/427) in the 9–20 years old age group, 2.32% (45/1942) in the 21–40 years old group and 4.90% (36/732) in the 41–77 years old group. By comparing the positivity rates of anti-*Toxoplasma* IgG antibody in the general population and psychiatric patients in different age groups, the results showed that the positivity rate of anti-*Toxoplasma* IgG antibody in the general population and psychiatric patients was significantly different between the 21–40 years old age group (*p* < 0.05) and 41–77 years old age group (*p* < 0.01) (Fig. 1).

**Figure 2:**
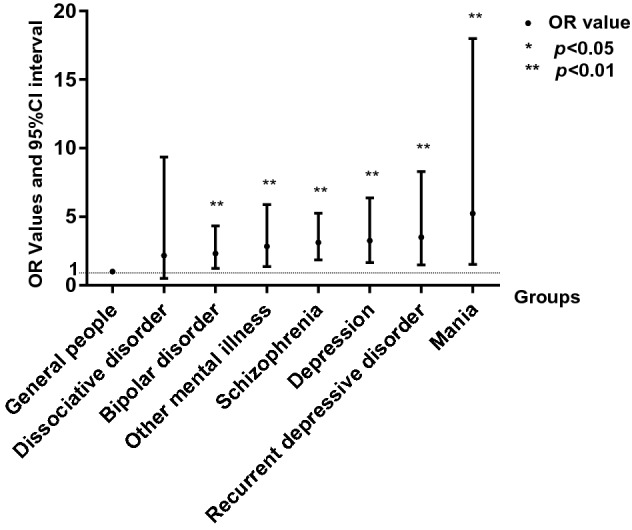
Comparison of anti-*Toxoplasma* IgG antibody-positivity rates in different groups. Compared with general people, the values of different types of psychiatric disorders are dissociative disorder (OR = 2.170 95% CI 0.504–9.350 *p* = 0.299), bipolar disorder (OR = 2.327 95% CI 1.249–4.337 *p* = 0.008), schizophrenia (OR = 3.136 95% CI 1.864–5.275 *p* = 0.000), depression (OR = 3.466 95% CI 1.740–6.906 *p* = 0.000), recurrent depressive disorder (OR = 3.160 95% CI 1.398–7.142 *p* = 0.006), mania (OR = 5.149 95% CI 1.501–17.659 *p* = 0.009), and others (OR = 2.821 95% CI 1.364–5.837 *p* = 0.005).

A large number of studies have shown that patients with certain types of mental illness, such as schizophrenia, have a higher infection rate. This study compared various types of mental illness with the general population and found that compared to the general population, depression, recurrent depressive disorder, schizophrenia, and mania patients had a higher positivity rate of anti-*Toxoplasma* IgG antibody (Fig. 2). It is noteworthy that the positivity rate of anti-*Toxoplasma* IgG antibody was highest in patients with mania and recurrent depressive disorder.

## Discussion

*T. gondii* infection is widespread around the world, recent studies have shown that the seroprevalence of anti-*Toxoplasma* antibody ranges from 10 to 80%. The seroprevalence of *Toxoplasma* infection in China is relatively low, ranging from 2.3% to 35.6% among different populations and geographic regions^8^. Numerous studies have found a higher serological prevalence of *T. gondii* infection in psychiatric patients^9–11^, and research on the relationship between *T. gondii* infection and psychiatric disorders has become a hot topic. China has a population of 1.4 billion, and the latest research^12^ shows that the weighted 12-month prevalence of mental disorders (excluding dementia) in China is 9.3% (95% CI 5.4–13.3), and the weighted lifetime prevalence is 16.6% (95% CI 13.0–20.2). Considering the large number of patients with mental illness in China, research on the epidemiological status of *T. gondii* infection and the association between mental illness and *T. gondii* infection is particularly important and necessary.

The results of the present study showed that the anti-*Toxoplasma* IgG and IgM antibodies in psychiatric patients and the general population were both at low levels in the study region^8,13,14^.

The positivity rate of anti-*Toxoplasma* IgG antibody in psychiatric patients was higher than that in the general population, which is similar to a previous study in Weihai, Shandong Province, between 2011 and 2013^13^. The results indicate that psychiatric patients in the study area should pay attention to the detection of *T. gondii* infection. There was no significant difference in the positivity rates of anti-*Toxoplasma* IgM antibody between the general population and psychiatric patients, which indicates that there is no difference in acute/recent infection of *T. gondii* among the general population and psychiatric patients in this study area. The seroprevalence of anti-*Toxoplasma* antibody in psychiatric patients suggests that *T. gondii* infections in psychiatric patients in the study area are mostly recessive or long-term. However, the results are inconsistent with some research. Wang's research^14^ in Zhejiang, China, showed that seropositivity rates of anti-*Toxoplasma* IgG antibodies and anti-*Toxoplasma* IgM antibodies were both significantly higher in psychiatric patients than in the nonpsychiatric control group, which may indicate geographical differences in the epidemiological status of *T. gondii* infection in psychiatric patients.

In this study, the positivity rate of anti-*Toxoplasma* IgM antibodies in both the general population and patients with mental illness was lower than the results published in China (Chen et al., 2019; Pan, M., et al., 2017; Chen, X., et al., 2019.). This value is especially lower than the research results in the nearby area (Weihai) (Cong et al., 2015). A large number of investigations have focused on the association between *T. gondii* infection and psychiatric disorders, but the results of these studies are inconsistent. Numerous studies have reported that *T. gondii* seropositivity is related to mental illnesses such as schizophrenia, bipolar disorder, generalized anxiety disorder, obsessive–compulsive disorder, suicide, aggression, and impulsivity^15–18^. However, others have failed to demonstrate significant associations between psychiatric disorders and toxoplasmosis^19,20^. Anti-*Toxoplasma* IgM antibody is a marker of acute/recent exposure, persistent infection, or reinfection^21^, while previous studies have focused on anti-*Toxoplasma* IgG antibody, which is a marker of lifetime exposure or latent *T. gondii* infection and has a higher positivity rate than IgM antibody. The latest research found that chronic *T. gondii* infection leads to cortical neurodegeneration and results in the interaction of CX3CL1, complement and microglia, thereby dividing and clearing degenerate neurons^22^. Therefore, the analysis of anti-*Toxoplasma* IgG antibody in this study is more meaningful.

After further analysis and comparison of different types of mental illness, the present study found that Mania, schizophrenia, bipolar disorder, depression and recurrent depressive disorder were all associated with positivity rates of anti-*Toxoplasma* antibodies, except for dissociative depressive disorder. The seroprevalence of *T. gondii* infection in patients with bipolar disorder was significantly different from that in the general population, which is consistent with newly published meta-analysis results^23,24^. However, some articles^7,19,25^ suggest that bipolar disorder is not associated with *T. gondii* infection. Therefore, the relationship between bipolar disorder and *T. gondii* infection is still controversial, and more research is needed.

A large amount of research suggests a link between *T. gondii* infection and schizophrenia, *T. gondii* becoming a potentially relevant aetiological factor in some cases of schizophrenia^26–28^, and recent studies suggest that *T. gondii* infection may be an underlying component of the pathophysiology of schizophrenia^29^, which is consistent with our study, but there are also some studies that reach the opposite conclusion. More rigorous studies are needed from epidemiological studies to mechanistic studies to confirm the relationship between schizophrenia and *T. gondii* infection.

Most research showed an absenc**e** of an association between depression and *T. gondii* infection^30–32^. However, a potential association between depression/recurrent depressive disorder and *T. gondii* infection was found in this study, and the same result was also found in Alvarado-Esquivel's study ^33,34^. More research on the relationship between *T. gondii* infection and depression is needed to explain the different conclusions. Furthermore, *T. gondii* infection affects the susceptibility and severity of depression in children, adolescents and pregnant women^35,36^, and patients with depression should pay attention to *T. gondii* infection. This study found a potential association between mania and *T. gondii* infection, but the current research on the relationship between mania and *T. gondii* infection is still insufficient to draw definitive conclusions, and more attention should be given to mania patients.

Existing research has shown that people who live in rural areas are at increased risk for toxoplasmosis^37^, but no consistent results were found in our study. Studies have found differences in the seroprevalence of anti-*Toxoplasma* antibody between males and females with psychosis^38^, which is inconsistent with our study. More research is needed to explain these phenomena. In the present study, psychiatric patients had the lowest positivity rate of anti-*Toxoplasma* IgG antibody in the 21–40 age group, indicating that this age group had the lowest risk of *T. gondii* infection, which may be because of the strong body and immunity in this age group.

The subjects included in this study are mainly from the southwestern regions of Shandong Province and parts of Henan and Jiangsu Province, with a population of approximately 40 million in the study area. Shandong Daizhuang Hospital is the largest psychiatric specialized hospital in Shandong Province; therefore, this study can accurately reflect the status of *T. gondii* infection in psychiatric patients in the study area and, to a certain degree, even east of China. The present study is a cross-sectional study, and cohort studies should be conducted in the future to better explain the internal connection of psychiatric disorders and *T. gondii* infection.

The present study mainly analysed *T. gondii* infection in psychiatric inpatients due to the difficulty in collecting basic data from the general population and outpatient mental patients. The population density in this study area is high, and the sample size included is relatively small. Because only the age information of the general population is available, more detailed personal information of the general population cannot be obtained for further analysis, and investigations with a larger sample size and different population groups should be performed to evaluate multiple influencing factors. It is widely believed that pregnant women should not have cats^39^, but research indicates that cat ownership in pregnancy or early childhood does not confer an increased risk of later adolescent psychotic experiences^40^. The extent to which cats are associated with *T. gondii* infection in patients with mental illness should be confirmed in future studies. Previous studies have suggested that anti-*Toxoplasma* antibody seropositivity persists throughout life^41^, but as research on anti-*Toxoplasma* antibodies increases and population-based analyses, some studies have suggested that persistent exposure to *T. gondii* is required for the maintenance of antibody levels^42^; therefore, anti-*Toxoplasma* antibody testing should be used as a routine test for assessing infection status and treatment monitoring in patients with mental illness.

At present, there have been many studies on the relationship between *T. gondii* infection and psychiatric disorders, and there are still many controversies and uncertainties. First, the results are very different. This may be because different people in different regions will have different results. Second, it is difficult to determine and explain the causal relationship between *T. gondii* infection and psychiatric disorders. From the perspective of aetiology and pathogenic mechanism, it is assumed that *T. gondii* infection will cause mental illness. It is assumed from an epidemiological perspective that patients with mental illness are susceptible to *T. gondii* infection due to poor health awareness or lower immunity. Therefore, research on the relationship between psychiatric disorders and *T. gondii* infection should be analysed in detail.

In summary, the seropositivity rate of *T. gondii* or the infection rate of *T. gondii* in the population in this region is maintained at a low level, but the seropositivity rate of *T. gondii* or the infection rate of *T. gondii* in patients with mental illness is maintained at a higher level than that in the general population. Age and different types of mental illness may be associated with *T. gondii* infection. More scientific and rigorous research from populations and laboratories is needed in our study region to determine the relationship between *T. gondii* infection and psychiatric disorders, especially mania, schizophrenia, depression, and recurrent depressive disorder.

## Data Availability

The datasets used and/or analysed during the current study are available from the corresponding author on reasonable request.
